# Genetic Variants at 20p11 Confer Risk to Androgenetic Alopecia in the Chinese Han Population

**DOI:** 10.1371/journal.pone.0071771

**Published:** 2013-08-26

**Authors:** Bo Liang, Chunjun Yang, Xianbo Zuo, Yang Li, Yantao Ding, Yujun Sheng, Fusheng Zhou, Hui Cheng, Xiaodong Zheng, Gang Chen, Zhengwei Zhu, Jun Zhu, Xuhui Fu, Tao Wang, Ying Dong, Dawei Duan, Xianfa Tang, Huayang Tang, Jinping Gao, Liangdan Sun, Sen Yang, Xuejun Zhang

**Affiliations:** 1 Institute of Dermatology and Department of Dermatology, No.1 Hospital, Anhui Medical University, Hefei, Anhui, China; 2 Key Laboratory of Dermatology, Anhui Medical University, Ministry of Education, China, Hefei, Anhui, China; 3 State key Laboratory Incubation Base of Dermatology, Anhui Medical University, Hefei, Anhui, China; Central China Normal University, China

## Abstract

**Background:**

Androgenetic alopecia (AGA) is a well-characterized type of progressive hair loss commonly seen in men, with different prevalences in different ethnic populations. It is generally considered to be a polygenic heritable trait. Several susceptibility genes/loci, such as *AR/EDA2R, HDAC9* and 20p11, have been identified as being involved in its development in European populations. In this study, we aim to validate whether these loci are also associated with AGA in the Chinese Han population.

**Methods:**

We genotyped 16 previously reported single nucleotide polymorphisms (SNPs) with 445 AGA cases and 546 healthy controls using the Sequenom iPlex platform. The trend test was used to evaluate the association between these loci and AGA in the Chinese Han population. Conservatively accounting for multiple testing by the Bonferroni correction, the threshold for statistical significance was P ≤3.13×10^−3^.

**Results:**

We identified that 5 SNPs at 20p11 were significantly associated with AGA in the Chinese Han population (1.84×10^−11^≤P≤2.10×10^−6^).

**Conclusions:**

This study validated, for the first time, that 20p11 also confers risk for AGA in the Chinese Han population and implicated the potential common genetic factors for AGA shared by both Chinese and European populations.

## Introduction

Androgenetic alopecia (AGA) is a common disease that is characterized by a distinctive pattern of progressive hair loss from the scalp. The proportion of affected males increases steadily with age, and its high prevalence in older men suggests that this form of hair loss may be a normal consequence of aging [1], [2]. The prevalence of AGA is different among races. The prevalence of AGA in Chinese males is lower than in Caucasians at all ages [3], [4].

The etiology of AGA was first described by Hamilton, including two essential components: genetic predisposition and hormone dependency [5], [6]. So far, several genetic factors, such as the androgen receptor (*AR*)/the ectodysplasin A2 receptor (*EDA2R*) [7]–[10], histone deacetylase 9 (*HDAC9*) [11] and 20p11 [12], [13], have been identified as being associated with this disorder. The present study aims to investigate whether the known susceptibility loci for the European populations are also associated with AGA in the Chinese Han population by the investigation of 8 SNPs at *HDAC9* [11], 5 SNPs on 20p11 [13] and 3 SNPs at the *AR/EDA2R* locus [9], [10].

## Materials and Methods

### Samples and DNA extraction

This investigation involved samples from 445 affected cases and 546 controls. All participants were males of Chinese Han descent (Table 1). The hair status of all participants was assessed by dermatologists according to the Hamilton/Norwood (HN) classification system. Affected men were <30 years of age with AGA grades IV–VII or <40 years with AGA grades V–VII. The controls comprised 546 unaffected male controls aged >60 years. Blood samples were collected in tubes containing disodium ethylene diamine tetraacetic acid (EDTA) as an anticoagulant and stored at −80°C until extraction. Genomic DNA was extracted from peripheral blood lymphocytes using Flexi Gene DNA kits (Qiagen, Hilden, Germany) according to standard procedures and diluted to working concentrations of 20 ng/μl for the replication study of the findings in the European population. All the samples were obtained after written informed consent. The study was approved by the Ethics Committee of Anhui Medical University and conducted according to Declaration of Helsinki Principles.

**Table 1:** Summary of AGA cases and controls.

Characteristic	Cases a	Controls b
**Total number**	445	546
**Age (years)**	28.38±4.43	65.72±4.67

**Note:**

a Effected men were <30 years of age with AGA grades IV–VII or <40 years with AGA grades V–VII.

b The controls comprised unaffected male controls aged >60 years.

### Genotyping

Genotyping was performed in multiplex reactions using the MassArray system and a Sequenom Compact MALDI-TOF device (Sequenom Inc., San Diego, CA, U.S.A.) at the Key Lab of Dermatology, Ministry of Education, Anhui Medical University, China. For the association analysis, the minor allele frequency (MAF) was ≥1% with P ≥0.05 for Hardy-Weinberg equilibrium in the controls, and SNPs with call rates higher than 95% in cases or controls were used for the SNP quality criteria.

### Statistical analysis

For the association testing, the Armitage trend test was used to detect allelic and genotypic effects using Plink 1.07 software (Harvard, Boston, MA, USA). The power was calculated using the power Fisher exact test in SPSS (SPSS version 13.0). In total, 16 SNPs were genotyped in 445 cases and 546 controls. Conservatively accounting for multiple testing by the Bonferroni correction, the threshold for statistical significance was P≤3.13×10^−3^.

## Results

Overall, we genotyped 16 SNPs in this study, which was performed in 445 AGA cases and 546 controls of Chinese Han descent using the Sequenom MassArray system. As shown in Table 2, the 3 SNPs (rs1385699, rs2497911, rs5919393) of the *AR/EDA2R* locus, which shows significant association with AGA in European populations, were monomorphic in the Chinese population. The 8 SNPs of the *HDAC9* locus, which reported susceptibility to AGA from a previous GWAS in European populations, showed no significance in the Chinese Han Population. After the initial analysis using the Armitage trend test, 5 SNPs (rs2180439, rs1998076, rs6113491, rs6137444 and rs201571) were significantly associated with AGA, exceeding the threshold of statistical significance for association (P<3.13×10^−3^).These five AGA susceptibility SNPs identified in the Chinese population are associated with one genetic locus (20p11). The SNP rs2180439 showed a stronger association (OR  = 1.92, P = 1.84×10^−11^) than the other four SNPs. Furthermore, controlling for the genetic effect of the SNP rs2180439 by using conditional logistic regression analysis also abolished the association of the other four SNPs (P>0.05, Table 3). These findings indicate that the other four SNP alleles are correlated with the SNP rs2180439 and that the association of the other four SNPs is mainly driven by the SNP rs2180439. The difference in the distribution of the rs2180439 genotype between the AGA cases and controls was significant (P = 1.29×10^−10^) (Table 4).

**Table 2:** Case-control association analysis for SNPs within AR/EDA2R, HDAC9 and 20p11 in 445 Han Chinese patients with AGA and 546 controls.

SNP	Sample^a^		locus	Position^b^	European						Chinese Han							
	Cases	Controls			A^c^/B Allele	MAF^d^ (Case/Control)	Risk allele	Allelic P	Risk Allelic OR(95%CI)	Ref	A^c^/B Allele	MAF^d^ (Case/Control)	Genotypes^e^		Risk Allele	Allelic P^f^	Risk Allelic OR(95%CI)	Power(%)
													Cases	Controls				
rs6137444	445	546	20p11	21733639	C/T	0.269/0.391	T	2.20×10^−**10**^	1.74(1.37–2.21)	[13]	C/T	0.272/0.401	34/174/237	96/246/204	T	1.68×10−9	1.79 (1.49–2.17)	98
rs2180439	444	540	20p11	21801100	C/T	0.293/0.452	T	2.67×10^−**15**^	1.82(1.45–2.30)		C/T	0.271/0.417	29/183/232	108/234/198	T	1.84×10−11	1.92(1.59–2.33)	99
rs1998076	445	540	20p11	21828045	A/G	0.292/0.448	G	7.73×10^−**15**^	1.9(1.50–2.41)		A/G	0.270/0.411	29/182/234	102/240/198	G	5.28×10−11	1.89(1.56–2.27)	99
rs201571	443	546	20p11	21961514	C/T	0.298/0.44	T	1.21×10^−**12**^	1.72(1.36–2.17)		T/C	0.428/0.324	77/225/141	72/210/264	T	2.10×10−6	1.56(1.30–1.87)	97
rs6113491	445	546	20p11	22005415	A/C	0.641/0.488	A	1.13×10^−**13**^	1.66(1.33–2.08)		A/C	0.425/0.313	81/216/148	66/210/270	A	2. 82×10−7	1.62(1.35–1.95)	98
rs3852255	445	546	HDAC9	18832972	T/C	0.045/0.030	T	1.45×10^−**1**^	1.54(0.87–2.72)	[11]	T/C	0.136/0.143	10/101/334	6/144/396	C	6.59×10−1	1.06(0.82∼1.37)	70
rs13230142	445	534	HDAC9	18848034	A/G	0.030/0.013	A	3.10×10^−**2**^	2.37(1.05–5.37)		A/G	0.117/0.118	8/88/349	6/114/414	G	9.39×10−1	1.01(0.77∼1.33)	90
rs12056282	443	540	HDAC9	18848728	C/T	0.043/0.022	C	2.90×10^−**2**^	1.98(1.05–3.75)		C/T	0.135/0.144	11/98/334	6/144/390	T	5.67×10−1	1.08(0.83∼1.39)	95
rs756853	442	534	HDAC9	18856525	G/A	0.496/0.380	G	4.65×10^−**6**^	1.61(1.30–1.98)		A/G	0.397/0.410	68/215/159	90/258/186	G	5.59×10−1	1.05(0.88∼1.27)	92
rs2249817	445	540	HDAC9	18862536	G/A	0.475/0.411	G	8.90×10^−**6**^	1.59(1.29–1.95)		A/G	0.300/0.267	39/189/217	42/204/294	A	1.02×10−1	1.18(0.97∼1.44)	82
rs10247184	445	546	HDAC9	18879472	T/C	0.002/0	T	3.34×10^−**1**^	–		T/C	0.123/0.137	9/91/345	18/114/414	T	3.28×10−1	1.14(0.88∼1.49)	90
rs10252945	441	528	HDAC9	18891375	A/C	0.286/0.256	A	1.97×10^−**1**^	1.17(0.92–1.47)		A/C	0.231/0.222	19/166/256	36/162/330	A	6.11×10−1	1.06(0.85∼1.31)	52
rs17350355	444	546	HDAC9	18895028	A/G	0.441/0.354	A	5.81×10^−**4**^	1.44(1.17–1.78)		A/G	0.389/0.412	68/209/167	102/246/198	G	2.87×10−1	1.10(0.92∼1.32)	36
rs1385699	445	546	AR/EDA2R	65741711	C/T	0.113/0.292	T	1.60×10^−**5**^	3.23(1.86–5.59)	[9]	T/T	1.000/1.000	445	546	–	NA	NA	NA
rs5919393	445	546	AR/EDA2R	66742082	C/T	0.046/0.218	T	6.00×10^−**7**^	5.82(2.73–12.41)		T/T	1.000/1.000	445	546	–	NA	NA	NA
rs2497911	445	546	AR/EDA2R	66544165	A/C	0.038/0.283	C	2.30×10^−**4**^	–	[10]	C/C	1.000/1.000	445	546	–	NA	NA	NA

Note:

a, Total samples (445 cases and 546 controls), some samples failed to genotype in the experiment;.

b, In bp. NCBI build 36.3;.

c, Minor allele;.

d, MAF =  Minor allele frequency;.

e, Numbers shown correspond to the following genotypes: AA/AB/BB (A = minor allele; B =  major allele).

f, the threshold for statistical significance was P≤3.13×10^−3^.

**Table 3:** Association and conditional logistic analysis for rs2180439, rs6137444, rs1998076, rs201571 and rs6113491 in 445 cases and 546 controls.

SNP	CHR	Position^a^	Risk Allele	Risk Allele Frequency		Allelic P	OR(95%CI)	Condition rs2180439	
				Case	Control			P	OR(95%CI)
rs2180439	20	21801100	T	0.729	0.417	1.84×10^–11^	1.92(1.59–2.33)	–	–
rs6137444	20	21733639	T	0.728	0.401	1.68×10^–9^	1.79(1.49–2.17)	7.22×10^–1^	1.09(0.70–1.67)
rs1998076	20	21828045	G	0.73	0.411	5.28×10^–11^	1.89(1.56–2.27)	7.43×10^–1^	1.14(0.53–2.44)
rs201571	20	21961514	T	0.428	0.324	2.10×10^–6^	1.56(1.30–1.87)	9.89×10^–2^	1.19(0.97–1.47)
rs6113491	20	22005415	A	0.425	0.313	2. 82×10^–7^	1.62(1.35–1.95)	5.38×10^–2^	1.26(0.99–1.55)

**Note:**

a, In bp. NCBI build 36.3.

**Table 4:** Genotypic effects analysis for rs2180439 in the AGA patients and controls.

		Cases (n = 445)	Controls (n = 546)	OR (95%CI)	P
**Genotype**	**CC**	29(6.53%)	108(20%)	Reference	1.29×10^−10^
	**TC**	183(41.22%)	234(43.33%)	2.91(1.85–4.58)	
	**TT**	232(52.25%)	198(36.67%)	4.36(2.78–6.85)	

## Discussion

This study is the first to investigate the contribution of the major AGA susceptibility loci *AR/ EDA2R*, 20p11 and *HDAC9* to the development of AGA in the Chinese Han population.

In this study, we confirmed that the susceptibility locus 20p11 for AGA in the Chinese Han population, which suggested the existence of common genetic factors shared for AGA in diverse ethnic populations. Because a gene contributing to the development of AGA would be expected to be expressed in the human scalp, Hillmer AM et al quantified the gene expression of the 20p11 locus and the expression of the closest paired box 1 (*PAX1*) gene [13]. The results showed that *PAX1* was expressed at very high levels in the scalp skin. Although the *PAX1* gene is more than 100 kb away from the 20p11–associated LD block, the expression data might suggest that *PAX1* confers the AGA-relevant effect at this locus and that a regulatory variant within the associated LD block may modulate its expression through long-range control. However, demonstration of the influence of the chromosome 20p11.22 locus on the transcription of the *PAX1* gene requires further investigation.

For the *HDAC9* locus, the statistical power calculations showed that we had >80% power to detect 5 SNPs (rs13230142, rs12056282, rs756853, rs2249817 and rs10247184) associated with AGA, whereas other 3 SNPs (rs3852255, rs10252945 and rs17350355) had low power (≤70%), with SNP rs17350355 having the lowest power (36%). For those SNPs that had low power to detect an association, we could not confirm that these SNPs lacked an association with AGA because we might lack the power to detect a true association. Larger sample sizes will help improve the power and ensure the correct conclusion with respect to whether these SNP are associated with AGA. For those SNPs that had >80% power to detect an association, we confirmed that these SNPs are not associated with AGA; however, we do not deny that *HDAC9* may be a risk factor for AGA because there could be different functional variants between Chinese and Caucasian populations or different LD patterns between the markers and hidden functional variants. For the *AR/EDA2R* locus, our genotype data showed that the three SNPs (rs1385699, rs2497911 and rs5919393) of the *AR/EDA2R* locus that show significant association with AGA in European populations were monomorphic in the Chinese population. These results showed that there is genetic heterogeneity among different populations. Genotyping other known SNPs in these region according to HapMap data will help elucidate the basis for potential ethnicity-related disparities in the AGA association for previously reported loci (*HDAC9* and *AR/EDA2R*) among different populations, which will provide a better understanding of the genetic basis of AGA.

Taken together, our study provided strong genetic evidence for the hypothesis that 20p11 might influence the susceptibility to AGA in both the Chinese Han and European populations, although the role of the 20p11 locus in the pathogenesis of AGA remains unclear. Further study using fine mapping and a functional study are warranted to investigate its exact role in the pathogenesis of AGA.
